# Gesundheitskompetenz und Gesundheitsverhalten – Einblicke in ein sich ausdifferenzierendes Forschungs- und Handlungsfeld für Public Health

**DOI:** 10.1007/s00103-025-04016-6

**Published:** 2025-02-14

**Authors:** Susanne Jordan, Maike Buchmann, Julika Loss, Orkan Okan

**Affiliations:** 1 Abteilung Epidemiologie und Gesundheitsmonitoring, Robert Koch-Institut, Gerichtstraße 27, 13347 Berlin, Deutschland; 2 TUM School of Medicine and Health, Department of Health and Sport Sciences, Technische Universität München, München, Deutschland

**Keywords:** Gesundheitskompetenz, Gesundheitsverhalten, Food Literacy, Physical Literacy, Behavioural and Cultural Insights, Health literacy, Health behaviour, Food literacy, Physical literacy, Behavioural and cultural insights

## Abstract

Das Forschungs- und Handlungsfeld „Gesundheitskompetenz und Gesundheitsverhalten“ differenziert sich zunehmend aus. Allgemeine Gesundheitskompetenz (aGK) ist etabliert, mit dem Fokus auf bevölkerungsweite Studien. Spezifische Gesundheitskompetenzen (sGK) zum Gesundheitsverhalten bieten themenbezogene Ansatzpunkte für Interventionen und Public-Health-Strategien.

Für die aGK und auch sGK aus den Bereichen Ernährung und Bewegung/körperliche Aktivität gibt es verschiedene Konzepte, Definitionen und Messinstrumente, die sich in Bezug auf die Handlungsebenen und Anwendungsbereiche der Gesundheitskompetenz unterscheiden.

Die meisten Studien zeigen eine positive Assoziation zwischen Gesundheitskompetenz (GK) und verschiedenen Gesundheitsverhalten. Eine höhere GK geht häufiger mit einem verbesserten gesundheitsförderlichen Verhalten einher. Dies gilt für die aGK wie auch für die sGK zur Ernährung und Bewegung. In einigen Studien wurde für bestimmte Verhaltensweisen kein Zusammenhang gefunden, in anderen nur für bestimmte Gruppen, was auch auf die unterschiedlichen Messinstrumente und Untersuchungskontexte zurückzuführen sein kann. Dies verweist auf die Bedeutung, stets auch die Wechselwirkung von Verhalten und Verhältnissen zu betrachten, um die Passung zwischen Individuum und den alltäglichen Anforderungen beim Umgang mit Gesundheitsinformationen zu verbessern.

Der Ansatz der „Behavioural and Cultural Insights“ (BCI) kann Erkenntnisse zur Förderung der GK im Hinblick auf verschiedene Gesundheitsverhalten liefern, zu individuellen Barrieren und Förderfaktoren, die sich aus Lebenslagen und Rahmenbedingungen ergeben und die soziale Praxis berücksichtigen. BCI und GK ergänzen sich und haben das Potenzial, Strategien zur Verbesserung des Gesundheitsverhaltens effektiver und zielgerichteter zu gestalten.

## Abstract (en)

The research and action field of health literacy and health behaviour is increasingly differentiating. General health literacy is established and focuses on population-based studies. Specific health literacy for health behaviour offers topic-related starting points for interventions and public health strategies.

There are various concepts, definitions and measurement instruments for general health literacy and specific health literacy in the areas of nutrition and physical activity. These differ in terms of the levels of action and areas of application of health literacy.

Most studies show a positive association between health literacy and various health behaviours. Higher health literacy is more often associated with improved health-promoting behaviour. This applies to both general as well as specific health literacy regarding nutrition and exercise (physical activity). Some studies found no correlation for certain behaviours, while others only found correlations for certain groups, which may be due to the different measuring instruments and research contexts. This points to the importance of always considering the interaction between behaviour and circumstances in order to improve the fit between the individual and the everyday demands of dealing with health information.

The behavioural and cultural insights (BCI) approach can provide insights into how to promote health literacy with regard to various health behaviours, individual barriers and facilitators that arise from life circumstances and conditions, and that take social practice into account. BCI and health literacy complement each other and have the potential to make strategies for improving health behaviour more effective and targeted.

## Einleitung

Gesundheitskompetenz beschreibt den Umgang mit Gesundheitsinformationen als Grundlage von individuellen gesundheitsrelevanten Entscheidungen [1]. Unabhängig davon, ob gesundheitsrelevante Entscheidungen bewusst oder impulsgesteuert getroffen werden, führen sie häufig zu konkreten Gesundheitsverhalten [2]. Mit Gesundheitsverhalten sind konkrete Verhaltensweisen gemeint, welche die eigene Gesundheit positiv beeinflussen können (bspw. körperliche Aktivität, Ernährung, Schlafverhalten, Früherkennungsuntersuchungen oder Impfungen). Damit wird die Stärkung von Gesundheitskompetenz relevant für die Förderung des Gesundheitsverhaltens [3].

Für die Förderung von Gesundheitskompetenz ist zu berücksichtigen, dass diese kontextabhängig ist und deswegen als ein „relationales Konzept“ bezeichnet wird [1]: Auf individueller Ebene umfasst Gesundheitskompetenz Fähigkeiten, Motivation, Wissen, soziale Praktiken und Selbstwirksamkeit [1]. Auf organisationaler und struktureller Ebene bezieht sich Gesundheitskompetenz auf komplexe situative Anforderungen, um gesundheitsrelevante Entscheidungen, z. B. für ein Gesundheitsverhalten, zu treffen [4]. Es wird angenommen, dass sich Gesundheitskompetenz auf gesundheitsbezogene Einstellungen in der motivationalen und volitionalen Phase der Entscheidungsfindung und damit auf das Gesundheitsverhalten auswirkt [5]. Dem breiten, mehrdimensionalen Konzept von Gesundheitskompetenz folgend, geht die Förderung von Gesundheitskompetenz im Hinblick auf ein gesundheitsförderliches Gesundheitsverhalten über ein enges Verständnis von Gesundheitsbildung und verhaltensorientierter Kommunikation hinaus und zielt auf kontextuelle, politische und soziale Einflussfaktoren [6].

Mit Gesundheitskompetenz liegt ein Konstrukt vor, das die gesundheitliche Handlungs- und Kompetenzebene zum Umgang mit Wissen und Information, um gesundheitsrelevante Entscheidungen und Gesundheitsverhalten zu beeinflussen, adressiert. Somit setzt sich Gesundheitskompetenz klar von Gesundheitswissen und Gesundheitsverhalten ab und füllt gleichzeitig die Lücke aus, die auf dem Spektrum von Wissen zu Gesundheitsverhalten existiert. Somit ist Gesundheitskompetenz als Konstrukt zwischen diesen beiden einzuordnen. Die wissenschaftstheoretische Auseinandersetzung mit dem Konstrukt [7] wie auch die Frage, ob Gesundheitskompetenz ein Mediator zwischen sozioökonomischen (z. B. Bildung) bzw. psychosozialen Faktoren (z. B. Selbstwirksamkeit) und gesundheitsbezogenen Faktoren wie dem Gesundheitsverhalten ist [8], haben erst begonnen.

Der Begriff Gesundheitskompetenz bezieht sich in der Regel auf die „allgemeine Gesundheitskompetenz“. Allgemeine Gesundheitskompetenz beschreibt themen- und kontextübergreifende Fähigkeiten im Umgang mit Gesundheitsinformationen und wird auch als generische oder generelle Gesundheitskompetenz bezeichnet. Im Unterschied zur allgemeinen Gesundheitskompetenz beziehen sich spezifische Gesundheitskompetenzen auf bestimmte Handlungsfelder, zum Beispiel auf konkrete Gesundheitsverhalten wie Ernährung und Bewegung, bestimmte Erkrankungen oder digitale Gesundheitsinformationen [1]. Neben dem Umgang mit Gesundheitsinformationen beinhalten die spezifischen Gesundheitskompetenzen teilweise weitere Kompetenzbereiche, die für das Verhalten wesentlich sind [1]. Exemplarisch seien hier die Bewegungs- oder Steuerungskompetenz im Bereich der Förderung körperlicher Aktivität genannt [9].

Die zunehmenden Erkenntnisse zu Gesundheitskompetenz und verschiedenen Gesundheitsverhalten sind Anlass, in diesem Beitrag einen Einblick in den aktuellen Forschungsstand hierzu zu geben. Neben Konzepten und Erkenntnissen zu allgemeiner Gesundheitskompetenz und Gesundheitsverhalten werden exemplarisch spezifische Gesundheitskompetenzen aus dem Bereich Ernährung und Bewegung vorgestellt. Den Abschluss bildet die Vorstellung des Konzepts „Behavioural and Cultural Insights“ und seiner Schnittmengen mit Gesundheitskompetenz im Hinblick auf die Förderung des Gesundheitsverhaltens.

## Allgemeine Gesundheitskompetenz und Gesundheitsverhalten

### Gesundheitskompetenz und Gesundheitsverhalten im Erwachsenenalter

Das Konzept der allgemeinen Gesundheitskompetenz hat sich in den letzten Jahrzehnten national und international in Forschung, Praxis und Politik etabliert und gilt als relevant für die Förderung des Gesundheitsverhaltens [6, 10, 11]. Das Gesundheitsverhalten kann dabei im Kontext von Aktivitäten zur Gesundheitsförderung (z. B. als Settingmaßnahme) als auch zur Prävention (z. B. individueller Rauchausstieg) oder in der Krankenversorgung (z. B. Inanspruchnahme von Nachsorgeterminen) adressiert werden. Diese Gesundheitskompetenz beschreibt themen- und kontextübergreifende Fähigkeiten im Umgang mit Gesundheitsinformationen und eignet sich besonders, um auf Bevölkerungsebene Bedarfe zur Förderung der Gesundheitskompetenz zu identifizieren und ihre Verteilung und Entwicklung in der Bevölkerung zu beobachten, insbesondere im Hinblick auf vulnerable Gruppen [12].

Gemessen wird die allgemeine Gesundheitskompetenz in der Regel in Form von (subjektiven) Selbsteinschätzungsinstrumenten wie dem Fragebogen des „Europäischen Health Literacy Survey“ (HLS-EU‑Q) [13] oder des „Health Literacy Questionnaire“ (HLQ) [14]. Bei diesen Instrumenten gibt die ausfüllende Person beispielsweise an, wie leicht bzw. schwierig es für sie ist, Informationen zu finden und zu verstehen (z. B. Informationen darüber, wie sie psychisch gesund bleiben kann). Das Messergebnis bildet die Passung zwischen den externen Anforderungen und den Fähigkeiten des Individuums ab [15, S. e29]. Dabei weisen die Selbsteinschätzungsinstrumente einen gewissen Interpretationsspielraum auf: Die Einschätzung einer Testperson, dass eine Aufgabe „schwierig“ sei, beruht zum einen darauf, dass die betreffende Person selbst nicht über die erforderlichen Kompetenzen verfügt, um die Aufgabe zu lösen. Zum anderen kann die Schwierigkeit der Aufgabe auch objektiv begründet sein, beispielsweise durch hohe Anforderungen oder komplexe Rahmenbedingungen. Auch sozial erwünschtes Antwortverhalten oder Selbstüberschätzung können die Aussagekraft der Ergebnisse einschränken. Leistungsbezogene (objektive bzw. performanzbasierte) Messinstrumente hingegen liefern objektivere Informationen durch die Testung auf Gesundheitswissen oder gesundheitsbezogene Lese- und Rechenfähigkeit. Beispiele dafür sind der „Newest Vital Sign Test“ (NVS), der „Rapid Estimate of Adult Literacy in Medicine“ (REALM) und der „Test of Functional Health Literacy in Adults“ (TOFHLA) [15, 16]. Diese Instrumente kommen in verschiedenen Studien zum Einsatz, um den Zusammenhang mit Gesundheitskompetenz und Gesundheitsverhalten zu erfassen, eignen sich aber nicht, um Gesundheitskompetenz mehrdimensional abzubilden, oder für den Einsatz in bevölkerungsweiten Studien. Daher sind Selbsteinschätzungsinstrumente trotz der oben genannten Limitationen für Bevölkerungsstudien praktikable und geeignete Messinstrumente.

In Deutschland konnten Studien mit umfassenden Selbsteinschätzungsinstrumenten, insbesondere dem HLS-EU‑Q, in den letzten Jahren zeigen, dass die Hälfte oder etwas mehr als die Hälfte der Bevölkerung Schwierigkeiten hat, mit Gesundheitsinformationen umzugehen [17–20]. Besonders betroffen sind Bevölkerungsgruppen mit niedrigem Sozialstatus, niedriger Bildung oder finanzieller Deprivation [17–20]. Daten aus verschiedenen Ländern legen ebenso nahe, dass Individuen mit höherer Gesundheitskompetenz eine Tendenz zu einem gesünderen Lebensstil im Hinblick auf den Obst- und Gemüsekonsum und körperliche Aktivität aufweisen [11, S. 184–185].

Zwischen allgemeiner Gesundheitskompetenz und Gesundheitsverhalten zeigt sich in vielen Studien eine positive Korrelation [17, 20, 21]. Zumeist werden der Umfang der körperlichen Aktivität, des Sporttreibens, Menge oder Häufigkeit des Obst- und Gemüsekonsums sowie Konsum bzw. Konsummenge von Tabak- und Alkohol untersucht. Hier zeigen sich studienspezifische Unterschiede: Je nach Studienpopulation, Messinstrument zur Gesundheitskompetenz bzw. dem Gesundheitsverhalten oder Analysemethoden sind in einigen Studien bzw. für bestimmte Teilpopulationen keine Assoziation zu beobachten [17, 20]. Im Hinblick auf die Absicht einer Verhaltensänderung, die oft das Ziel von Interventionen ist, hat sich Gesundheitskompetenz als moderierende Variable bei körperlicher Aktivität gezeigt [22].

Im Hinblick auf das Gesundheitsverhalten fokussieren sich Forschung und Interventionspraxis häufig auf die individuelle Gesundheitskompetenz. Public-Health-Strategien wie der „Nationale Aktionsplan Gesundheitskompetenz“ [10] oder die Strategie der Weltgesundheitsorganisation (WHO) zur Bekämpfung von nichtübertragbaren Erkrankungen legen den Schwerpunkt anders [6]. Sie empfehlen den Fokus nicht nur auf die individuelle Ebene zu legen, sondern Gesundheitskompetenz als Teil von Verhältnisprävention zu verstehen, damit Normen und Kulturen sowie organisatorische und politische Strukturen verändert werden (soziale Praxis), die die Entwicklung von Gesundheitskompetenz in der Bevölkerung maßgeblich beeinflussen [6].

### Gesundheitskompetenz und Gesundheitsverhalten im Kindes- und Jugendalter

Gesundheitskompetenz hat sich mittlerweile auch im Bereich der Kinder- und Jugendgesundheit als wichtiges Interventionsziel sowohl für die Förderung der körperlichen als auch der psychischen Gesundheit etabliert. Die WHO betont seit 10 Jahren die besondere Rolle der Schule für die Stärkung der Gesundheitskompetenz [12]. In Deutschland werden solche Empfehlungen insbesondere durch den „Nationalen Aktionsplan Gesundheitskompetenz“ und die „Allianz für Gesundheitskompetenz in der Schule“ ausgesprochen [10]. Konzeptionell orientieren sich Gesundheitskompetenzansätze für Kinder und Jugendliche an den Gesundheitskompetenzkonzeptionen für Erwachsene, die den Umgang mit Gesundheitsinformationen für gesundheitsbezogene Entscheidungen adressieren. Adaptierungen und Erweiterungen der Gesundheitskompetenzansätze für Kinder und Jugendliche zu spezifischen Gesundheitskompetenzen liegen insbesondere zu digitalen, psychischen und auf das Gesundheitsverhalten bezogenen Gesundheitskompetenzen vor [23, 24].

Basierend auf diesen Konzepten wurden in den letzten Jahren unterschiedliche Instrumente entwickelt [25, 26], die ab dem Grundschulalter eingesetzt werden können [27]. Diese reichen von (subjektiven) Selbsteinschätzungsinstrumenten bis zu leistungsbezogenen (objektiven bzw. performanzbasierten) Messverfahren, sowohl für allgemeine als auch spezifische Varianten von Gesundheitskompetenz. Derzeit finden Fragebogenverfahren, die teilweise oder ganz auf dem Fragebogen des „European Health Literacy Survey“ (HLS-EU‑Q) [28, 29] oder dem „Digital Health Literacy Instrument for Adolescents“ (DHLI) [30, 31] basieren bzw. verwandte Verfahren weite Verbreitung. Auch ethnografische Methoden werden zunehmend angewendet [32].

Die Studien deuten auf einen Zusammenhang zwischen der Gesundheitskompetenz von Kindern und Jugendlichen und ihrem Gesundheitsverhalten hin [33–36]. Eine Evidenzsynthese für das Europäische Schulnetzwerk kommt zu dem Schluss, dass eine höhere Gesundheitskompetenz häufiger mit gesundheitsförderlichen Verhaltensweisen einhergeht (z. B. in Bezug auf Ernährung, Sport und Bewegung, Substanzkonsum, Sexualverhalten, Ruhe und Schlafverhalten) [37]. Dabei gibt es Unterschiede je nach Art des Gesundheitsverhaltens, insbesondere der Substanzkonsum scheint sich von Ernährung und Bewegung zu unterscheiden, wie beispielsweise die bundesweite Befragung zur „Gesundheitskompetenz von Jugendlichen“ (GeKoJu) zeigt: Gesundheitskompetenz ist mit dem täglichen Konsum von Obst und Gemüse assoziiert. Niedrigere Level in behavioralen, affektiven und konativen (absichtsorientierten) Dimensionen der Gesundheitskompetenz erhöhen die Chance, körperlich inaktiv zu sein und zu rauchen. Bei riskantem Alkoholkonsum spielen andere Faktoren (wie Alter oder familiärer Wohlstand) eine wichtigere Rolle als allgemeine Gesundheitskompetenz [34]. In einer Studie zur Gesundheitskompetenz bei Jugendlichen an Schulen in Hessen sind mittlere und geringe digitale Gesundheitskompetenz mit weniger körperlicher Aktivität, nichttäglichem Obstkonsum und täglichem Konsum von zuckerhaltigen Getränken assoziiert [31]. Die repräsentative Befragung von Grundschulkindern in der 4. Schulklasse zeigt [27], dass eine höhere Gesundheitskompetenz den stärksten Prädiktor für regelmäßiges Zähneputzen sowie Obst- und Gemüsekonsum darstellt [38]. Soziale Ungleichheiten stellen auch im Kindes- und Jugendalter ein wiederkehrendes Phänomen dar, denn die Ausprägung von Gesundheitskompetenz folgt einem sozialen Gradienten [27], welcher sich auch bei der positiven Assoziation zwischen Gesundheitskompetenz und Gesundheitsverhalten im Jugendalter findet [34].

## Spezifische Gesundheitskompetenzen an den Beispielen Ernährung und Bewegung

Spezifische Gesundheitskompetenzen fokussieren auf Bedarfe hinsichtlich bestimmter Gesundheits- und Krankheitsthemen, um passgenaue Interventionen zu entwickeln [39]. Konzepte spezifischer Gesundheitskompetenz sollen Anforderungen spezifischer gesundheitsrelevanter bewusster oder unbewusster Entscheidungssituationen oder Alltagsroutinen abbilden und ergänzen Erkenntnisse zu allgemeiner Gesundheitskompetenz. Für den Bereich des Gesundheitsverhaltens wurden je nach Verhaltensweise theoriebasiert eigene Dimensionen und Kompetenzbausteine für die spezifische Gesundheitskompetenz definiert, auch als Grundlage für die Entwicklung von spezifischen Messinstrumenten. Konzepte spezifischer Gesundheitskompetenzen gibt es in den Bereichen Ernährung und Bewegung, aber auch Rauchen, Alkoholkonsum, Schlafverhalten, Impfverhalten etc. [40–45]. Im Folgenden werden exemplarisch 2 Bereiche spezifischer Gesundheitskompetenzen vorgestellt, für die in Forschung und Praxis ausdifferenzierte Konzepte, darauf aufbauende Erhebungsinstrumente und eine Interventionspraxis, zumindest in Modellprojekten, vorliegen: Ernährung und Bewegung.

### Food Literacy – Ernährungsbezogene Gesundheitskompetenz

Für den Ernährungsbereich gibt es verschiedene Konzepte und Definitionen, die sich hinsichtlich der Handlungsebenen, Anwendungsbereiche und Domänen der Ernährungskompetenz und auch der Benennung der Gesundheitskompetenz unterscheiden [40, 46–48]. *Funktionelle ernährungsbezogene Gesundheitskompetenz* (*Nutrition Literacy*) bezieht sich auf das Verständnis von Informationen über Lebensmittel oder Ernährung [48]. Sie umfasst vereinzelt zusätzlich interaktive und kritische Fähigkeiten [49]. *Food Literacy* wird auch als Ernährungskompetenz bzw. ernährungsbezogene Gesundheitskompetenz bezeichnet. Sie geht über Wissen und Verstehen hinaus und beinhaltet praktische Fähigkeiten, z. B. Mahlzeitenplanung, Lebensmittelauswahl und -zubereitung [48, 50], manchmal ergänzt um Kontextfaktoren (z. B. Verfügbarkeit gesunder Lebensmittel) oder psychologische Faktoren (z. B. motivationale, volitionale und behaviorale Aspekte). Food-Literacy-Konzepte für junge Menschen betonen „Food Systems“ („Ernährungssystem“: Gesamtheit der Lebensmittelversorgung und der gesellschaftlichen Ernährungsnormen) und soziale Gerechtigkeit [51–53] bzw. soziale Aspekte zu Ernährung und Körperbild [53]. Die Förderung der Ernährungskompetenz soll die Gesundheit auf Bevölkerungsebene verbessern und gesundheitliche Ungleichheit reduzieren [46]. Damit ist Ernährungskompetenz relevant für Public Health, was die steigende Zahl an Publikationen zu diesem Konzept erklärt [47].

Die Ausdifferenzierung von Ernährungskompetenz spiegelt sich in der Messung von Ernährungskompetenz wider [54, 55]. Die Nutrition Literacy erfassenden Instrumente sind performanzbasierte Messinstrumente, z. B. die validierten Instrumente „Nutrition Literacy Scale“ (NLS) [56] und „Newest-Vital-Sign“(NVS)-Test [57] (letzterer wird auch für die Erfassung allgemeiner funktionaler Gesundheitskompetenz eingesetzt). NLS und NVS-Test messen performanzbasiert die Fähigkeit, gedruckte Informationen zu verstehen. Beispielsweise sollen beim NVS-Test Befragte Nährwertangaben auf einer Eiscremepackung verstehen und 6 Fragen dazu beantworten, z. B. wie viele Kalorien sie beim Verzehr des gesamten Packungsinhalts zu sich nehmen würden. Von diesen 6 Fragen beantwortete knapp ein Drittel der Teilnehmenden in einer repräsentativen Befragungsstudie von Erwachsenen in Deutschland (*N* = 1974) höchstens 3 Fragen richtig [58], ähnliche Ergebnisse zeigten sich in einer bundesweiten Befragung in Österreich [59]. Das breitere Konzept Food Literacy wird mithilfe von multidimensionalen Selbsteinschätzungsinstrumenten erfasst [55], z. B. mit der validierten „Self-Perceived-Food-Literacy“(SPFL)-Skala. Sie umfasst 29 Fragen, aus deren Antworten ein Gesamtscore sowie 8 Subscores gebildet werden, die folgende Bereiche umfassen: „Selbst zubereiten“, „Gemeinsam essen“, „Gesund haushalten“, „Wahl der Vorräte“ „Widerstehen können“, „Smart snacken“, „Mahlzeiten planen“ und „Gesund vergleichen“ [58, 60]. Eine höhere Food Literacy ist mit einem häufigeren Obst‑, Gemüse- und Fischkonsum sowie mit mehr Selbstkontrolle und weniger Impulsivität assoziiert [60]. Eine erste Studie für Deutschland fand eine höhere Ernährungskompetenz bei Frauen, steigendem Alter, höherem Bildungsabschluss und höherem Einkommen [58, 61]. Mahlzeitenplanung und Vergleich von Nährmittelangaben bewerteten die Befragten als am schwierigsten [58].

Für Kinder und Jugendliche existieren ebenfalls unterschiedliche Instrumente, die Nutrition Literacy, Food Literacy oder beide Konstrukte messen, und für unterschiedliche Altersgruppen validiert wurden [51, 62], z. B. „Nutrition Health Literacy Scale for Children“ (NHL‑C) [63, S. 27 ff.] und „Food Literacy Questionnaire for schoolchildren“ (FLQ-sc) [64]. Es gibt Hinweise auf einen positiven Einfluss von verschiedenen Teilaspekten der Food Literacy auf die Nahrungsaufnahme und Ernährungsgewohnheiten von Jugendlichen [65]. Die meisten Nutrition- und Food-Literacy-Instrumente für Kinder und Jugendliche werden allerdings als zu wenig umfassend bzw. als zu wenig lebensphasenspezifisch kritisiert oder erheben nur Teilaspekte von Food Literacy [51, 62, 65].

Ernährungskompetenz liefert Ansatzpunkte auf individueller Ebene, um Wissen, Fähigkeiten und Ressourcen zu stärken, um sich in der Ernährungslandschaft zu orientieren, informierte Ernährungsentscheidungen zu treffen sowie nahrhafte Mahlzeiten herzustellen. Auf Bevölkerungsebene weist sie darauf hin, wo verhältnispräventive Public-Health-Strategien ansetzen sollten, z. B. Nährwertkennzeichnung (wie Nutri-Score) oder Gemeinschaftsverpflegung. Die vorhandenen bevölkerungsweiten Studien stellen dafür zentrale Befunde bereit (z. B. [58]).

### Physical Literacy – Bewegungsbezogene Gesundheitskompetenz

Für den Bereich körperliche Aktivität und spezifische Gesundheitskompetenz ist eine konzeptionelle Vielfalt zu beobachten. Gemeinsam ist den *Physical-Literacy-*Konzepten das Verständnis von einer Kompetenz, welche Bewegung im gesamten Lebensverlauf fördern soll und im Kindes- und Jugendalter eine Bildungsaufgabe darstellt [45, 66]. Übergreifend werden neben motorischen Basiskompetenzen (funktionelle körperliche Ebene), Wissen bzw. Verständnis eines aktiven Lebensstils (kognitive Ebene), Motivation und Selbstbewusstsein (affektive Ebene) als zentrale Elemente von Physical Literacy gesehen [67]. Ein weiteres Konzept wird als *bewegungsbezogene Gesundheitskompetenz* bezeichnet; es umfasst 3 Teilkompetenzen: Bewegungskompetenz, Steuerungskompetenz (Ausrichtung auf Wohlbefinden und Gesundheit) und Selbstregulationskompetenz (Motivation, Volition) [68, 69]. Neben personalen Komponenten werden in Konzepten der bewegungsbezogenen Gesundheitskompetenz auch Umgebungsfaktoren und soziale Teilhabe [70] berücksichtigt und das Konzept um die Forderung nach „Physical Literate Societies“ ergänzt [71].

Für die Erfassung von Physical Literacy liegen verschiedene Messinstrumente vor. Ein Mangel aller Messinstrumente besteht im Fehlen der sozialen Komponente, die der Physical Literacy inhärent ist [72]. Als umfassende Messinstrumente für Physical Literacy werden auf Basis von systematischen Übersichtsarbeiten das „Canadian Assessment of Physical Literacy“ (CAPL) sowie der „Passport for Life“ [45, 72] für Kinder empfohlen (für Erwachsene gibt es keine klare Empfehlung für ein Instrument [45]). Das Tool CAPL erfragt für einen Physical Literacy Score verschiedene Aspekte aus den Domänen „Physical Competence“, „Daily Behaviour“, „Knowledge and Understanding“ und „Motivation and Confidence“ [73]. Mit dem CAPL konnte in Studien ein positiver Zusammenhang zwischen Physical Literacy und körperlicher Aktivität bei Kindern von 8–12 Jahren gezeigt werden [74–76]. Die meisten Instrumente decken nur eine oder 2 Ebenen von Physical Literacy ab, wobei körperliche und affektive Kompetenzen eher erfasst werden als kognitive Kompetenz [45]. So erfasst ein Teil der Messinstrumente körperliche Kompetenzen, z. B. durch Aufgaben, die motorische Basisfertigkeiten, Balance, Kraft oder kardiovaskuläre Fitness erfordern, quantitativ mithilfe von Zeitmessung [72]. Ein anderer Teil der Messinstrumente erfasst affektive und/oder kognitive Aspekte von Physical Literacy, z. B. die „Children’s Physical Activity Self-Efficacy Scale“ oder die „Children’s Self-Perception of Adequacy in and Predilection for Physical Activity Scale“ [72].

Insgesamt gibt es bisher wenig Forschung zum Zusammenhang von Physical Literacy und körperlicher Aktivität bei Erwachsenen, vermutlich auch, da bisher nicht konsentiert ist, ob Bewegungsverhalten Teil des Physical-Literacy-Konzeptes selbst ist oder ein Outcome von Physical Literacy und damit eine außerhalb des Konstrukts liegende Variable darstellt [45, S. 1067]. Für Physical Literacy insgesamt sowie körperliche und motivationale, nicht aber kognitive Komponenten sind positive Zusammenhänge beschrieben [45, 77]. Im deutschsprachigen Raum wurden mithilfe validierter Lang- sowie Kurzinstrumente bei verschiedenen Bevölkerungsgruppen Zusammenhänge zwischen Komponenten bewegungsbezogener Gesundheitskompetenz und körperlicher Aktivität gezeigt [78].

Physical-Literacy-Interventionen zeigen in Studien auf mehrere für körperliche Aktivität und Gesundheit relevante Endpunkte positive Effekte [79] und verweisen damit auch auf Ansatzpunkte für verhältnispräventive Public-Health-Strategien.

## „Behavioural and Cultural Insights“ und Gesundheitskompetenz als Ansätze zur Förderung des Gesundheitsverhaltens

Bei der Untersuchung der Frage, wie Gesundheitskompetenz und Gesundheitsverhalten zusammenhängen, kann auch der interdisziplinäre Ansatz der „Behavioural and Cultural Insights“ (BCI) herangezogen werden. BCI umfassen empirische Erkenntnisse, die erklären, wie Menschen in ihrem Alltag gesundheitsrelevante Entscheidungen treffen und welche Faktoren ihr Gesundheitsverhalten beeinflussen. Der BCI-Ansatz weist viele Ähnlichkeiten mit bestehenden Public-Health-Ansätzen auf, stellt allerdings das Verhalten als zentrale Zielgröße in den Mittelpunkt und zielt darauf ab, individuelle und strukturelle Barrieren und Einflussfaktoren zu identifizieren, um daraus präventive Maßnahmen abzuleiten [80].

Der BCI-Ansatz bietet verschiedene Anknüpfungspunkte zur Gesundheitskompetenz, insbesondere im Hinblick auf die Analyse und Förderung des Gesundheitsverhaltens. Laut dem COM-B-Modell von Michie [81], das eine zentrale theoretische Grundlage des BCI-Ansatzes bildet, beeinflussen nicht nur Fähigkeiten (C = Capabilities) und Motivation (M = Motivation), sondern auch strukturelle und soziale Faktoren (O = Opportunity) das Verhalten (B = Behaviour) [82]. Zum einen zeigen sich Parallelen zur Gesundheitskompetenz: So bedarf Gesundheitskompetenz nicht nur Gesundheitswissen und Fähigkeiten, sondern auch Anpassungen der Umwelt, z. B. durch organisationale Gesundheitskompetenz. So lenken beide Ansätze die Aufmerksamkeit darauf, dass Gesundheitsverhalten nicht nur von Wissen und Kompetenzen, sondern insbesondere von den sozialen Kontexten, dem Umfeld und den konkreten Lebenswelten geprägt wird. Zum anderen lässt sich Gesundheitskompetenz als eine Fähigkeit (Capability) im COM-B-Modell einordnen. Damit kann BCI dazu beitragen, den Zusammenhang zwischen Gesundheitskompetenz und Gesundheitsverhalten besser zu verstehen. Umfassende Definitionen von Gesundheitskompetenz schließen auch motivationale Aspekte ein, ein Bereich, der im COM-B-Modell einen eigenständigen Faktor (Motivation) darstellt. Diese Überschneidung sollte bei Analysen zum Gesundheitsverhalten berücksichtigt werden.

Die Einbeziehung von BCI-Studien in die Gesundheitskompetenzforschung eröffnet neue Perspektiven, da für BCI auch unbewusste gesundheitsrelevante Entscheidungen untersucht werden. Der BCI-Ansatz adressiert die aus der Prävention und Gesundheitsförderung bekannte Beobachtung, dass Menschen trotz vorhandenen Wissens ihr Verhalten nicht ändern (der sogenannte Intention-Action-Gap) und hat so das Potenzial, strukturelle Barrieren zu identifizieren, an denen Gesundheitskompetenzförderung ansetzen kann.

Der Ansatz „Behavioural and Cultural Insights“ wird in Public Health verstärkt verfolgt [82, 83], andererseits kontrovers diskutiert. Das mag daran liegen, dass der Ansatz ursprünglich als „Behavioural Insights“ durch die Verhaltensökonomie geprägt wurde, mit einer engen Ausrichtung auf psychologische Verhaltens- und Entscheidungsmuster und im Hinblick auf sogenannte Nudging-Maßnahmen, die durch kleine Umgebungsänderungen oder Anreize erwünschte Verhaltensänderungen erleichtern [84, 85]. Die WHO hingegen betont, dass sich Verhaltenswissenschaften von einer individuellen, verhaltensbezogenen Perspektive deutlich erweitern müssen hin zu einem umfassenderen, systemischen Ansatz [86]. Sie verdeutlicht diesen Wandel durch die Ergänzung von „Behavioural Insights“ um den Begriff „cultural,“ dadurch wird das soziokulturelle Umfeld adressiert [82]. Damit wird deutlich, dass BCI wie auch Gesundheitskompetenzförderung nur dann erfolgreich soziale Chancengleichheit adressieren können, wenn sie im Sinne von „New Public Health“ Rahmen- und Lebensbedingungen verstehen und im Sinne von Verhältnisprävention positiv gestalten helfen, um das Gesundheitsverhalten in der Bevölkerung zu verbessern.

## Fazit

Das Forschungs- und Handlungsfeld „Gesundheitskompetenz und Gesundheitsverhalten“ differenziert sich zunehmend aus. Studien zu allgemeiner Gesundheitskompetenz und Gesundheitsverhalten eignen sich in besonderem Maße für die vergleichende Beobachtung auf Bevölkerungsebene. Untersuchungen zu spezifischen Gesundheitskompetenzen hinsichtlich einzelner Gesundheitsverhalten liefern Informationen zu konkreten Ansatzpunkten für Interventionen. Die Erhebungsinstrumente ergänzen sich demnach. Allgemein zeigen die zumeist als Querschnittsstudien durchgeführten Studien eine positive Assoziation zwischen Gesundheitskompetenz und verschiedenen Gesundheitsverhalten. Dies gilt sowohl für die allgemeine Gesundheitskompetenz als auch für die hier näher untersuchten spezifischen Gesundheitskompetenzen zur körperlichen Aktivität und Ernährung. Eine höhere Gesundheitskompetenz geht häufiger mit einem verbesserten gesundheitsförderlichen Verhalten ein. In manchen Studien finden sich nicht bei allen untersuchten Gesundheitsverhaltensweisen diese Zusammenhänge und manchmal nur für bestimmte Gruppen. Das kann teilweise auf verschiedene Studiendesigns und Messinstrumente zurückzuführen sein, aber auch auf unterschiedliche Kontexte wie sozioökonomische Bedingungen und soziale Normen. Letztere verweisen auf den Bedarf, stets auch die Wechselwirkung von Verhalten (Mensch) und Verhältnissen (Struktur) zu untersuchen und im Rahmen von Interventionen zu berücksichtigen. Gesundheitskompetenz als Determinante von Gesundheitsverhalten ist in individuelle Lebenslagen und soziale Kontexte eingebettet. Der Ansatz „Behavioural and Cultural Insights“ kann für die Förderung von Gesundheitskompetenz im Hinblick auf verschiedene Gesundheitsverhalten wissenschaftliche Erkenntnisse liefern, zu persönlichen Barrieren und Förderfaktoren, die auf Lebensumstände und Rahmenbedingungen zurückgehen und die soziale Praxis berücksichtigen. BCI und Gesundheitskompetenz ergänzen sich und haben das Potenzial, effektivere und zielgerichtetere Strategien zur Verbesserung des Gesundheitsverhaltens zu entwickeln.
